# Inequalities in Severe Maternal Morbidity and Mortality in High-Income Countries: Patterns, Drivers, and Pathways to Action

**DOI:** 10.1097/AOG.0000000000006298

**Published:** 2026-04-23

**Authors:** Nicola Vousden, Elie Azria, Dorothea Geddes-Barton, Catherine Deneux-Tharaux

**Affiliations:** National Perinatal Epidemiology Unit, Nuffield Department of Women's and Reproductive Health, University of Oxford, Oxford, and Clinical and Biomedical Sciences, University of Exeter, Exeter, United Kingdom; and Université Paris Cité et Université Sorbonne Paris Nord, Inserm, INRAE, Center for Research in Epidemiology and Statistics (CRESS), Obstetric Perinatal and Pediatric Life Course Epidemiology (OPPaLE), iWISH, and Maternité Hôpital Paris Saint Joseph, FHU Prem’Impact, Paris, France.

## Abstract

Inequalities in severe maternal morbidity (SMM) and mortality in high-income countries are persistent, socially patterned, and evident across multiple dimensions, including socioeconomic deprivation, race and ethnicity, and migration status. These inequalities are not fully explained by individual clinical risk factors but arise from the interaction of structural disadvantage, intermediate social conditions, and health systems. Many determinants of risk are established before pregnancy; however, variation in access to care, quality of care, and responsiveness to symptoms during pregnancy and childbirth can either mitigate or exacerbate vulnerability. Identifying social and structural determinants, ensuring equitable access to care, providing culturally responsive care, and promoting timely, unbiased clinical decision making are essential components of clinician efforts to reduce inequalities in SMM and mortality.

## Abstract 2

Health care professionals play a crucial role in identifying and mitigating social exposures and in advocating for change to decrease inequalities in maternal morbidity and mortality.

Every day in 2023, more than 700 women died of preventable causes related to pregnancy and childbirth.^1^ For every woman who dies, an estimated 100 women will experience severe maternal morbidity (SMM).^2,3^ There is no single definition of SMM, but it frequently refers to a composite of serious, potentially life-threatening complications during pregnancy or the early postpartum period.^4^ Striking inequalities in maternal mortality are observed between high- and low-income countries, with more than 90% of maternal deaths in 2023 occurring in low- and lower-middle–income countries.^1^ Inequalities in maternal mortality and SMM are also significant between subgroups within high-income countries. These inequities occur across a range of dimensions or characteristics as shown in Figure 1.

**Fig. 1.**
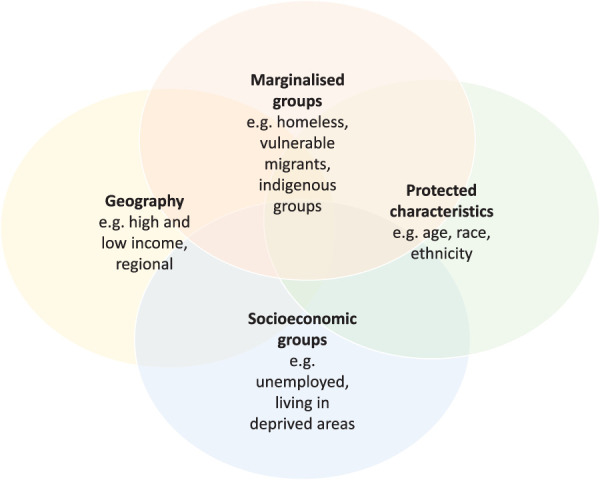
Overlapping dimensions of inequalities in maternal mortality and severe morbidity. Adapted from “Health Equity Assessment Tool (HEAT): executive summary” by Public Health England.^9^ Available at https://gov.uk/government/publications/health-equity-assessment-tool-heat/health-equity-assessment-tool-heat-executive-summary. Contains public sector information licensed under the Open Government Licence v3.0.

*Vousden. Inequalities in Maternal Morbidity and Mortality. Obstet Gynecol 2026*.

Inequalities in maternal mortality and SMM are pervasive and persistent across contexts.^5^ For example, in the United Kingdom, rates of maternal mortality have been twofold to sevenfold higher for Black women compared with White women since consistent detailed reporting began in the early 2000s.^6^ However, describing inequalities by a single characteristic is likely to inadequately convey the scale of inequity and injustice because women often experience multiple distinct exposures such as race, socioeconomic position, and migration status. The effects of these intersecting characteristics are not just additive but have complex interactions that influence power and privilege.^7,8^ This is represented by the overlapping circles of Figure 1.^9^

*Inequalities* can be defined as unfair and avoidable differences in health across the population and between different subgroups within society.^10,11^ From an ethical perspective, inequalities are neither natural nor biologically determined but arise from avoidable and unjust social conditions.^11^ Therefore, understanding and addressing maternal health inequalities not only is a matter of improving individual outcomes but also lessens the economic and organizational burden on health care systems and raises fundamental questions of social justice. Finally, reducing maternal health inequalities is a key lever in combating the intergenerational transmission of these inequalities.

The objectives of this article are to give a clinical perspective on the extent and dimensions of inequalities in SMM and mortality in high-income countries, to examine the mechanisms that explain how contexts of social disadvantage lead to an increased risk, and to review the proposed potential actions for clinicians.

## EXTENT AND DIMENSIONS OF INEQUALITIES IN SEVERE MATERNAL MORBIDITY AND MORTALITY

Socioeconomic disadvantage, for example, unemployment, can be measured at the neighborhood level or for individuals. Multiple high-income countries, including the United States, Europe, and Australia, showed a clear and consistent pattern: Both women living in socioeconomically deprived neighborhoods and women experiencing individual-level socioeconomic disadvantage face increased risks of SMM and maternal mortality.^12^ This association persists across different definitions of disadvantage, analytic models, and health care systems, suggesting that socioeconomic inequalities continue to undermine maternal health even in resource-rich environments. It is important to note that inequalities in SMM increase as deprivation increases. This gradient persists whether deprivation is measured at the area level (Index of Multiple Deprivation or Socio-Economic Indexes for Areas)^3,13,14^ or individual level^15^ and is independent of individual characteristics. However, individual factors may better clarify the role of specific disadvantage factors that may be obscured by composite area-level measures of deprivation.^16^

Migration status represents another important area of inequality.^17^ Europe is the region of the world that has seen the largest increase in the proportion of migrants within its population over the past 30 years and where the absolute number of migrants is currently the highest.^18^ Thus, a large proportion of births occur among non–native-born women, more than one-quarter in France and the United Kingdom.^18,19^ Evidence from Europe indicates that migrant women often experience higher risks of severe maternal outcomes than their native-born counterparts. In France, the maternal mortality rate is 2.5 times higher in migrant women from sub-Saharan Africa compared with French-born women, a gap that has remained unchanged over the last four reports (2007–2018).^20^ In the Netherlands, a similar gap is found for migrant women from Surinam/Dutch Antilles.^21^ In Spain, the Human Development Index score of the country of maternal origin has been shown to be significantly associated with maternal mortality (a decrease of 0.01 in Human Development Index score significantly increases the risk of maternal mortality by 2.4%).^22^ Beyond the characteristics of the country of origin, women with other dimensions of migration status such as no legal status and language barriers have the highest excess risk of SMM,^23^ revealing other sources of heterogeneity within the broad group of migrant women and insight into potential mechanisms.

In contrast, studies from the United States and Australia have not observed an overall excess risk among migrants. These divergent patterns highlight the heterogeneity hidden within the broad category of migrant women. When analyses disaggregate migrants by region of birth, a more consistent picture emerges: Women originating from sub-Saharan Africa, Latin America, the Caribbean, and many parts of Asia face substantially higher risks of SMM and mortality than women born in the host country.^17^ Interpreting inequalities in migrant women requires close attention to who is included in the native-born reference group. For example, in the United States and Australia, Black women^24^ and Indigenous women,^25,26^ respectively, face markedly higher risks of severe maternal outcomes. However, in most studies of migrant health, these groups would be included in the reference category, against which health outcomes of migrant women are compared. This classification may underestimate the true inequities affecting migrant communities and hide other significant internal inequities for nonmigrant women.

*Race* can be defined as a group of people connected by common descent or origin, and *ethnicity* is defined as membership of a group, ultimately of common descent or having common national or cultural tradition.^27^ Racial and ethnic inequalities remain among the most widely reported and striking patterns in the epidemiology of SMM and mortality. In the United States, maternal mortality ratios for non-Hispanic Black women are three to four times higher than for White women, a disparity that has not narrowed over decades and persists across other demographics and obstetric characteristics.^28^ In the United Kingdom in 2021–2023, Black women were at 2.3 times greater risk and Asian women at 1.3 times greater risk of maternal death than White women.^29^ For SMM, Black African, other Black, and Bangladeshi women in the United Kingdom experienced the highest overall risk of SMM compared with White women, an effect that was not mediated by deprivation.^13^ Similarly, in the United States, Black, Hispanic, Asian/Pacific Islander, and American Indian women have higher rates of a composite of SMM compared with non-Hispanic White women,^30^ and better neighborhood environments and higher resources did not protect Black women from SMM in the same way as White women.^31^

Together, these patterns reveal that inequalities in SMM and mortality in high-income countries are multifactorial and exist through a range of sociodemographic characteristics. It is important to note that we describe the intersection between race and deprivation as interconnected and interrelated domains. There is comparatively limited robust research describing the complex intersecting effects of different characteristics on inequalities in maternal mortality and SMM, including more nuanced approaches and understanding how other social identities such as Indigenous identity, gender identity, disability, and migration status interact and influence experience, quality of care, and outcomes.^8,13,32–34^ Analytic methods such as mediation analysis, structural equation modeling, and mixed methods may help characterize the contribution of the different dimensions of social status.

## MECHANISMS UNDERLYING SOCIAL INEQUALITIES IN MATERNAL HEALTH

The literature describing the mechanisms that underly inequalities includes varying definitions of social exposure and methodologic approaches. Many frameworks can be applied to understand these mechanisms of action.^35^ This section uses the multilevel structures of the World Health Organization (WHO) Commission on Social Determinants of Health conceptual framework to describe the pathways linking social position to maternal morbidity and mortality.

## CONCEPTUAL FRAMEWORK

**Fig. 2.**
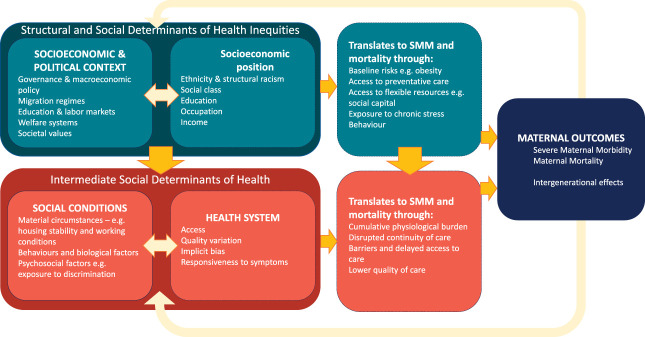
Examples of structural and intermediate determinants of health and how they influence severe maternal morbidity (SMM) and mortality.

Persistent social inequalities in SMM and mortality cannot be explained by individual clinical risk factors alone.^5^ Although conditions such as hypertension, obesity, diabetes mellitus, or advanced maternal age contribute to adverse outcomes, they do not account for the patterned distribution of risk observed across social groups.^13,36^ Instead, these inequalities emerge from a multilevel causal chain linking upstream structural determinants, intermediate social conditions, health system processes, and downstream clinical events.^11^ In this framework, maternal outcomes are not isolated biological occurrences but the result of cumulative exposures operating across the life course and throughout pregnancy. This is demonstrated in Figure 2, which applies the WHO conceptual framework to maternal health inequalities. The following sections describe how these structural determinants underpin inequalities in SMM and mortality, both directly and through influencing the intermediate social determinants and health system.

*Vousden. Inequalities in Maternal Morbidity and Mortality. Obstet Gynecol 2026*.

### Structural Determinants: The Upstream Production of Maternal Risk

At the most upstream level, social inequalities in SMM are rooted in structural determinants, including economic and social policies, migration regimes, housing and labor markets, educational systems,^37^ and systems of racial and gender stratification.^11^ These macrolevel forces shape women's health trajectories long before pregnancy begins, influencing the distribution of cardiometabolic risk factors such as obesity, access to preventive care, and exposure to chronic stress—all of which are directly implicated in major causes of SMM such as hypertensive disorders, hemorrhage, and thromboembolism.^38,39^

Socioeconomic disadvantage affects SMM through interconnected pathways. Economic precarity limits access to stable housing,^40^ adequate nutrition, and safe working conditions,^41^ contributing to higher prevalence and earlier onset of chronic conditions entering pregnancy. Residential segregation and neighborhood deprivation are associated with reduced access to high-quality obstetric services, including facilities equipped to manage obstetric emergencies. These structural conditions such as housing and working conditions influence not only baseline health status but also access to flexible resources such as knowledge, time, and social capital that may be critical when obstetric complications arise.^42^

Structural racism and restrictive migration policies such as tightening rules for people seeking asylum and restricted access to universal maternal care for undocumented migrant women further shape maternal risk by producing cumulative psychosocial stress and differential access to health care coverage and social protection.^23^ By the time pregnancy occurs, vulnerability to SMM is already socially patterned, making adverse obstetric events the downstream expression of long-standing structural inequality rather than isolated clinical failures.

### Intermediate Social Conditions: Translating Structure Into Experience of Health and Care

Structural inequalities become biologically and clinically relevant through intermediate social conditions that shape women's lived experience of health and health care.^11^ These include housing stability, employment circumstances, social support networks, exposure to discrimination or violence, and chronic psychosocial stress. These social conditions are embodied in and translated into differential vulnerability during pregnancy.

Chronic stress has been proposed as one important mediating pathway.^39^ Sustained exposure to financial strain, racial discrimination, or legal insecurity may contribute to cumulative physiologic burden, often conceptualized as allostatic load,^39^ reflecting repeated activation of stress-response systems over time.^43^ Elevated allostatic load has been associated with adverse pregnancy outcomes and hypertensive disorders in several studies, although causal pathways remain complex and incompletely understood.^39^ These mechanisms may help explain how long-standing social disadvantage contributes to cardiometabolic dysregulation, inflammatory processes, and vascular dysfunction implicated in SMM.

Intermediate social conditions also influence maternal outcomes through behavioral and health care pathways. Precarious employment, caregiving burdens, or unstable housing^44^ may delay prenatal care initiation and disrupt continuity of care.^45,46^ Education,^47^ language barriers,^23,48,49^ and limited health literacy constrain effective communication, informed decision making,^50^ and timely recognition of warning signs, potentially affecting the management of time-sensitive complications such as preeclampsia.^51^ It is important to note that these intermediate exposures cluster among women who are socioeconomically disadvantaged, racialized, or subject to restrictive migration regimes, concentrating risk within populations already shaped by upstream structural inequities.^23,52^

### Health System Mediation: Care as Both a Buffer and an Amplifier

Health care systems play a pivotal role in shaping how social disadvantage translates into SMM. Health systems may both mitigate and amplify social inequalities through differential access, variable quality of care, and institutional organization.

Barriers to access remain a critical mechanism. Maternal social vulnerabilities have been consistently associated with delayed^53^ or suboptimal prenatal care utilization and suboptimal childbirth care,^17,47^ even in settings in which the principle of universal access to health care prevails.^17,45^ Administrative complexity, insurance restrictions, and gaps in coverage may delay presentation or limit continuity of care, particularly for socioeconomically disadvantaged and migrant populations.

Quality of care across institutions represents another pathway. Research shows that Black and Hispanic women in the United States are more likely to deliver in hospitals with higher complication rates after standardizing for risk.^30,54^ This indicates that differential sorting of patients across hospitals with varying resources and performance levels may contribute to preventable disparities. Within clinical encounters, communication quality, responsiveness to reported symptoms, and clinical decision making may differ across social groups and contribute to differential care.^55^ Racial and ethnic disparities in SMM and mortality persist after clinical risk factors are accounted for.^36,56,57^ These findings suggest that processes of care may play a role.^58,59^ Women, especially from ethnic minority groups, have reported feeling judged or unheard or experiencing racist behaviors or culturally inappropriate care,^55,60^ and this is confirmed in many independent inquiries^6,61,62^ and qualitative and survey approaches.^63^ Such differences in communication may affect understanding of warning signs, adherence to follow-up recommendations, and engagement in shared decision making. There is a need to translate these complex experiences and quality of care into measurable outcomes to enable comparison and evaluation of progress.^8,64^

In maternal care, many life-threatening complications, particularly hypertensive emergencies, begin with subjective or nonspecific symptoms. Delayed evaluation or escalation of care contributes to SMM and mortality.^3^ When clinical judgment relies on discretionary assessment rather than strict algorithmic triggers, implicit biases may subtly influence the interpretation of symptoms and treatment decisions.^6,65^ Although rarely overt, this may contribute to patterned differences in monitoring, diagnostic testing, or escalation of care. Language discordance, limited health literacy, and power asymmetries within clinical encounters may further constrain patients' ability to advocate for themselves or to communicate symptom severity. These interpersonal dynamics do not imply intentional discrimination but rather highlight how institutional culture, time pressures, and cognitive heuristics may interact with social hierarchies to produce differential responsiveness. Such differences in recognition and management may contribute to persistent social inequalities in SMM.

## CLINICAL AND SERVICE LEVEL ACTIONS TO REDUCE INEQUALITIES IN SEVERE MATERNAL MORBIDITY AND MORTALITY

**Fig. 3.**
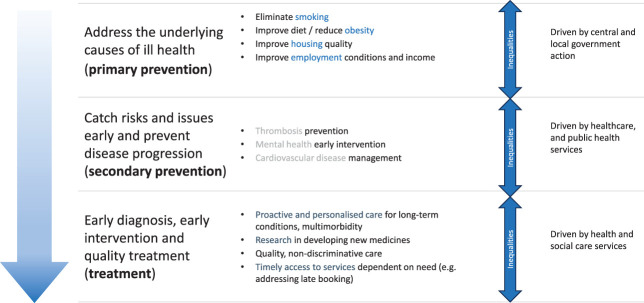
Identifying the level of actions to address inequalities in maternal health.

We have described structural and intermediate mechanisms of inequalities that require action at the level of the national and local governments (Fig. 3). There is growing evidence that policies to tackle structural determinants, including the U.S. Earned Income Tax Credit, expansion of public insurance,^66^ and cash transfers,^67^ may reduce inequalities in maternal and perinatal morbidity and mortality, but it is beyond the scope of this clinical perspective to comprehensively describe them. Health systems also play a critical role in mediating inequalities and mitigating their effects (Fig. 2). This can be directly through care and through support and advocacy to mitigate the effect of structural determinants. The remainder of this review therefore describes the evidence for actions that clinicians caring for obstetrics and gynecology patients may take as individuals and within health systems.

### Identifying and Responding to Social and Structural Determinants of Health in Clinical Care

National and professional guidance in several high-income countries recommends routine assessment of social and structural determinants of health during maternity care, including housing instability, financial insecurity, migration-related vulnerability, and exposure to violence.^3,68–70^ The specific factors that are identified, the timing of inquiry, and whether identification is supported by use of a standardized tool vary between settings. It is vital that this approach is trauma informed.^71^

At the individual level, identification of social and structural drivers of health provides clinicians an opportunity to refer or signpost to wider services, including welfare, community, and charity sector supports and wider health care services. Although this is often supported by local, regional, or national policy, there is limited evidence describing the influence of these policies on maternal mortality and SMM.^72^ Outside of maternity, research suggests that initiatives such as social prescribing and colocating welfare with health care can result in clinical and cost efficiencies.^73–75^

### Improve Experience of Care

Recognition of the sociocultural context of maternity care is important to women and health care service professionals in all regions of the world.^76^ Many countries have national policies to improve the delivery of culturally informed, respectful care^62,77^; these policies often focus on individual clinical change, for example, through cultural competency training programs. Specific culturally sensitive care training programs such as the one tested in the ORAMMA (Operational Refugee and Migrant Maternal Approach) project conducted in three European countries have proved efficient at improving midwives’ cultural competencies.^78^ However, the evidence of efficacy of specific approaches to reduce bias in maternity care is limited.^79–81^ Clinicians operate within complex health systems that have to facilitate equitable care. A recent interim report of a national maternity investigation in the United Kingdom has described how working environment, workload, workforce, culture, and leadership all underpin the ability of clinicians to provide safe care.^82^ These are likely critical to care that reduces inequalities, but this relationship remains poorly described. System-level interventions include person-centered care charters, which define and declare baseline expectations of respectful care, including an equity and intersectional lens in clinical training curricula,^8^ and systematically measuring experience^64^ and quality of care^8^ with relevant dedicated tools^83^ to evaluate the effect of these interventions.

*Vousden. Inequalities in Maternal Morbidity and Mortality. Obstet Gynecol 2026*.

### Improve Access to Care

At the service level, it may be possible to lower barriers to health care by including flexible appointment times and location and availability of transport^60,76^ and offering support such as vouchers for transport,^3^ interpreters,^84^ or timely support for maternity benefits when available.^84^

Experience and uptake of care can also be influenced by the model of care. Prioritizing relational continuity, through group care and continuity of midwifery care, can improve outcomes such breastfeeding, antenatal care uptake,^85,86^ and preterm birth in disadvantaged groups.^84–87^ However, their influence on reducing inequalities in SMM for women with disadvantage requires further research.^88,89^ Women experiencing intersecting disadvantage—including mental health conditions, substance use, or social instability—frequently need to navigate care across multiple services. Guidelines often recommend that maternity care be integrated with psychiatric, health, and social care workers to reduce the burden of care,^90^ but implementation of this type of integrated clinic frequently is challenged by siloed health care systems.^3,91^ Again, emerging evidence demonstrates potential positive effects of multidisciplinary care on perinatal outcomes and antenatal care attendance for migrant women,^84^ but the effect on SMM and mortality remains largely unknown.^92^

### Shape the Wider System

Health care professionals can play a key role in shaping how services are organized and delivered by supporting quality improvement initiatives that measure health equity and evaluating the effect of interventions on inequalities. The WHO framework also emphasizes a human rights approach^11^; for health care professionals, this can mean involving patients and communities in care planning and service design. For example, collaborative approaches to quality improvement in California using a toolkit for hemorrhage resulted in a significant reduction in SMM overall with the greatest benefit in Black women (9.0% vs 2.1% absolute rate reduction).^93^

At the system level, routine measurement and transparent reporting of disparities are fundamental to improving care and reducing inequalities. Confidential inquiries and national surveillance systems have demonstrated the value of disaggregated data in identifying preventable factors and institutional contributors to inequity.^48^ However, data on ethnicity, migration status, and socioeconomic position are inconsistently ascertained across high-income settings. For example, although the United Kingdom has a systematic approach to collect ethnicity within its health data, countries such as France, Denmark, and Germany do not record ethnicity in an effort to emphasize the principle of universality between individuals.^94^ As a consequence, disparities may remain unmeasured and unaddressed. Although policy-level decisions about measuring determinants of health are shaped by ethical, cultural, legal, and historical considerations, this constrains the ability of clinicians to identify inequities and to target resources proportionate to needs. However, clinicians can advocate for improved measurement in their systems.

## CONCLUSIONS

Inequalities in SMM and mortality in high-income countries arise from the interaction of structural disadvantage, lived social conditions, and health system responses. Although risk is socially patterned long before pregnancy begins, how health systems respond during pregnancy and childbirth can either mitigate or amplify these vulnerabilities. Health care professionals play a crucial role in providing care that identifies and mitigates social exposures, challenges inequitable systems, and advocates for change. Equity must be embedded as a core dimension of quality and safety of maternity care. This includes systematic identification of social determinants, equitable and flexible access to services, culturally responsive and respectful care, and timely, unbiased recognition and escalation of clinical concerns. Routine measurement and transparent reporting of disparities in outcomes should inform quality improvement efforts and service redesign. Because the dimensions along which inequalities are studied are often shaped by available data, future research must move to disentangle intersecting exposures and to rigorously evaluate structural and system-level interventions for their effect on equity.
